# Exploring the health benefits and functional properties of goat milk proteins

**DOI:** 10.1002/fsn3.3531

**Published:** 2023-06-27

**Authors:** Qausar Hamed ALKaisy, Jasim S. Al‐Saadi, Ali Khudhair Jaber AL‐Rikabi, Ammar B. Altemimi, Mohammad Ali Hesarinejad, Tarek Gamal Abedelmaksoud

**Affiliations:** ^1^ Department of Food Science, College of Agriculture University of Basrah Basrah Iraq; ^2^ Department of Dairy Science and Technology, College of Food Sciences University of AL‐Qasim Green Al Qasim Iraq; ^3^ Department of Food Processing Research Institute of Food Science and Technology Mashhad Iran; ^4^ Food Science Department, Faculty of Agriculture Cairo University Giza Egypt

**Keywords:** coprecipitates, goat milk, nutritional properties, proteins

## Abstract

Goat milk proteins are unique in their nutritional and functional properties and have become increasingly popular in recent years. A variety of methods have been studied for extracting and isolating these proteins, with coprecipitation being a particularly effective approach. Compared to cow milk proteins, goat milk proteins contain higher levels of certain amino acids such as tryptophan and cysteine, while maintaining similar nutritional properties. Additionally, they have superior functional properties, including better emulsifying and foaming properties, which make them an attractive option for developing new food products. Research has shown that goat milk proteins have several health benefits, including immunomodulatory effects, allergy management, anti‐inflammatory, and antioxidant effects, as well as antimicrobial and anticancer properties. They have the potential to be used as a treatment for autoimmune diseases, allergies, and other immune system disorders due to their ability to modulate the production of cytokines and other immune system components. Furthermore, their antimicrobial properties can help prevent the growth of harmful bacteria and reduce the risk of infection. Future research will focus on the potential of goat milk proteins as a functional food ingredient, their effects on gut health and microbiota, and their therapeutic potential for various health conditions. This research may lead to the development of new functional foods that promote health and prevent disease, and potentially pave the way for the use of goat milk proteins as a therapeutic agent for various health conditions.

## Graphical abstract

Future research on goat milk proteins will focus on their potential as a functional food ingredient, their effects on gut health and microbiota, and their therapeutic potential for various health conditions. This research may lead to the development of new functional food products that promote health and prevent disease. Additionally, it may pave the way for the use of goat milk proteins as a therapeutic agent for a range of health issues.

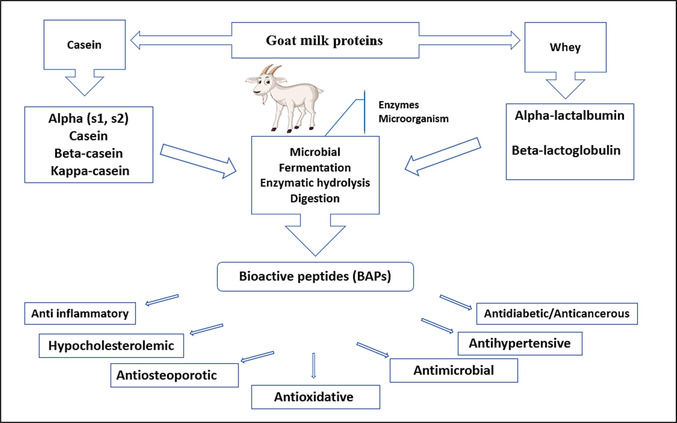

## 1 INTRODUCTION

The importance of goats in human nutrition has probably been recognized since the early stages of domestication. In fact, the first research papers on goat's milk published in The Lancet mainly emphasized its role in infant nutrition and discussed various advantages and disadvantages associated with it (Clark & García, 2017).

Tiwari et al. (2022) stated that goat's milk is a valuable source of nutrition, especially in regions where cow's milk is not easily accessible or commonly consumed. In 2018–2019, there were approximately 1.003 billion goats worldwide, of which approximately 203 million were identified as dairy goats, producing 15.26 million tons of milk annually. Asia accounts for more than half of global goat milk production, with India, Pakistan and Bangladesh making significant contributions (Nayik et al., 2022). The unique nutritional and functional properties of goat milk proteins have attracted attention (Van Leeuwen et al., 2020), including higher digestibility, buffering capacity and alkalinity compared to cow's milk (Saikia et al., 2022). Goat milk proteins have different ratios of caseins and whey proteins than cow's milk, resulting in different functional properties (Qin et al., 2021). Thus, goat milk proteins show promise for use in a variety of food products, especially for those who are sensitive to cow milk (Csapóné Riskó & Csapó, 2019).

The potential health benefits of consuming goat milk proteins have also been reported. Studies suggest that goat milk proteins may have a positive effect on the bioavailability of minerals, particularly iron and calcium (Lopez‐Aliaga et al., 2003). In addition, goat milk proteins have been shown to be less allergenic than cow milk proteins, making them a suitable alternative for individuals with cow's milk sensitivities (El‐Agamy, 2007). There is a growing interest in various markets for functional proteins for use in the food industry, which has led to an increased focus on improving the production and separation techniques of milk proteins with specific functions (Mati et al., 2017). One method of achieving this is through the production of coprecipitated proteins, which offer various benefits such as higher nutritional value and increased coagulability and functionality (Al‐Shamsi et al., 2018). Various methods have been developed to produce milk protein concentrates, each with its own unique structure and properties, making them ideal as food ingredients. Several techniques for the production and processing of coprecipitated proteins have already been established, and there is potential to discover new methods that could yield greater benefits for the use of coprecipitated proteins as commercial products in the food processing sector (Brodkorb et al., 2016). AlGhsyar et al. (2018) discussed the physicochemical and functional properties of protein coprecipitates obtained from camel's and goat's milk. The researchers found that the coprecipitates had high protein content and good functional properties, such as emulsifying and foaming abilities. They also observed that the goat milk coprecipitates had better solubility and thermal stability. The authors suggest that these coprecipitates could be used as functional ingredients in various food products. This study provides insight into the potential of camel and goat milk coprecipitates as a source of functional proteins for the food industry (AlGhsyar et al., 2018). Despite the promising potential of goat milk proteins, there is a need for a comprehensive review of their nutritional and functional properties. Such a review would help to identify areas for further research and improve our understanding of the potential benefits of consuming goat milk proteins and their derived products for human health and nutrition.

The main objective of this review is to achieve several goals. Firstly, it aims to provide a thorough overview of the nutritional and functional properties of goat milk proteins, including the different types of protein coprecipitates and the methods used for their preparation. Secondly, it aims to investigate the factors that influence the interaction between milk proteins and minerals and quantify these interactions. Thirdly, the nutrient profiling and functional properties of goat milk proteins will be investigated to assess their potential health benefits. Fourthly, it aims to evaluate the nutritional value of products derived from goat milk protein coprecipitates. Finally, it aims to identify areas where further research is needed to improve our understanding of the potential benefits of consumption of goat milk proteins and derived products for human health and nutrition.

## 2 REVIEW METHODOLOGY

**FIGURE 1:**
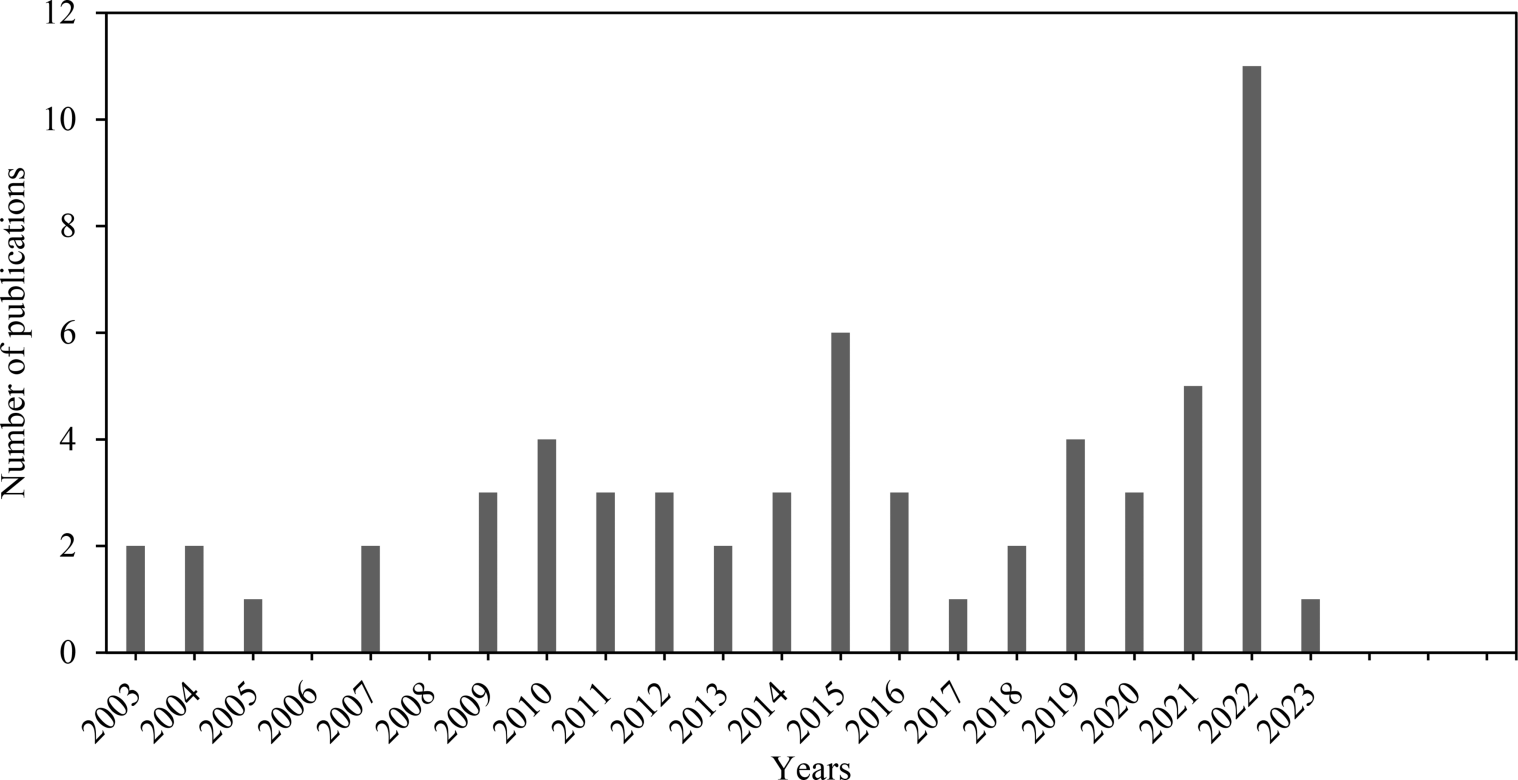
A comparative study of research papers published over time in Scopus and web of science databases, with a special emphasis on keywords associated with “Goat milk proteins, co‐precipitates, and nutritional properties.”

The researchers extensively searched two databases, Web of Science and Scopus, based in London, UK, to gather information on the topic of “goat milk”. They narrowed their focus to the subtopic of “food,” as shown in Figure 1, and chose Web of Science because of its collection of articles, indexed journals, and user‐friendly interface. Their initial search using keywords such as “goat milk proteins; co‐precipitates, nutritional properties of goat milk proteins” yielded 2064 articles, of which 1324 articles were published since 2013. Figure 1 shows a significant increase in the use of goat's milk in the food industry over the last 25 years. From 2003 to 2023, there was a noticeable increase in interest in goat's milk and in these years, there was a significant increase in publications, around five per year.

### 2.1 Extraction and isolation of goat milk proteins

Goat's milk contains different protein fractions such as casein, whey proteins, and minor proteins, each with unique properties and functions (Dhasmana et al., 2022). The extraction and isolation of these proteins from goat's milk is important for the development of functional foods and nutraceuticals (Dos Santos et al., 2022). In this article, we will discuss the methods of protein extraction and purification, the properties of different protein fractions, and the factors affecting protein yield and quality. Extraction and purification of proteins from goat's milk involve several steps, such as separation of fat and milk solids, protein precipitation, and chromatographic separation. The following are some commonly used methods for the extraction and purification of proteins from goat's milk:

1. Ultrafiltration: This method uses a membrane with a specific pore size to separate milk proteins based on their size and charge. Ultrafiltration can be used to isolate casein and whey proteins separately or together. This method is suitable for large‐scale production and can yield high‐quality protein isolates (Damar et al., 2020).
2. Acid precipitation: This method involves adjusting the pH of the milk to around 4.6, which causes the casein to precipitate out of the milk. The precipitated casein can be separated from the liquid whey fraction by centrifugation. This method is simple and inexpensive, but may not yield high‐quality protein isolates (Augustin et al., 2011).
3. Ion exchange chromatography: This method uses a resin with a charged surface to separate proteins based on their charge. The positively charged whey proteins can be separated from the negatively charged casein and other proteins. This method can produce high‐quality protein isolates but is expensive and time‐consuming (Tsakali et al., 2010).
4. Gel filtration chromatography: This method uses a column packed with beads of different pore sizes to separate proteins based on their size. Smaller proteins can pass through the beads, while larger proteins are trapped and eluted later. This method can be used to separate the different whey proteins and can provide high‐quality protein isolates (Neyestani et al., 2003).

Goat's milk contains several protein fractions, each with unique properties and functions. The following are the main protein fractions found in goat's milk:

1. Casein: Casein is a heterogeneous protein. It is made up of several fractions. It is a phosphoprotein present in milk as a micelle. Casein is relatively insoluble and forms a gel‐like structure when precipitated from milk. Casein is slowly digested in the stomach, making it an ideal source of sustained‐release protein (Park et al., 2007).
2. Whey proteins: Whey proteins make up about 20% of the total protein content of goat's milk. Whey proteins are a mixture of several proteins such as β‐lactoglobulin, α‐lactalbumin, immunoglobulins, and lactoferrin. Whey proteins are more soluble than casein and are quickly digested in the stomach, making them an ideal source of fast‐release protein (Hejtmánková et al., 2012).
3. Minor proteins: Goat's milk also contains several minor proteins such as lysozyme, lactoperoxidase, and serum albumin. These proteins have various functions, such as antimicrobial activity and regulation of the immune system. Minor proteins are present in small amounts in goat's milk, but they can be isolated and purified for specific applications (El‐Hatmi et al., 2015).

There are several factors that influence the protein yield and quality of goat's milk:

1. Breed of goat: The breed of goat is an important factor influencing the protein yield and quality of goat's milk. Different breeds of goats have different milk production and composition characteristics. Some breeds, such as Alpine and Saanen, have been shown to have higher protein yields than other breeds, such as Nigerian dwarf and pygmy goats. The protein content of goat's milk can also vary between breeds. For example, the protein content of Saanen goat's milk is generally higher than that of Nigerian dwarf goat's milk (Goetsch et al., 2011).
2. Nutrition: This is another important factor that has an impact on the protein yield and quality of goat's milk. The protein content of goat's milk is influenced by the protein content of the goat's diet. Goats fed a high‐protein diet will produce milk with higher protein content. In addition to protein, other nutrients such as energy, minerals, and vitamins are important for milk production and quality. A deficiency in any of these nutrients can adversely affect the protein yield and quality of goat's milk. The quality of the animal's diet plays an important role in determining milk quality. For example, dairy cows that consume high‐quality forage, such as fresh pasture or nutrient‐rich hay, tend to produce milk with higher levels of beneficial fatty acids such as omega‐3. Conversely, feeding too much grain without a proper balance can alter the fatty acid composition of milk, increasing saturated fats and reducing unsaturated fats. In addition, supplementing cows' diets with omega‐3 fatty acids or using feed additives such as probiotics can positively influence milk composition. Adequate mineral supplementation is also essential to ensure proper milk production and quality. However, it is important to note that the specific effects of nutrition on milk quality can vary depending on factors such as species, breed, and stage of lactation (Goetsch et al., 2011).
3. Stage of lactation: The composition and properties of goat's milk show significant variations at different stages of lactation, which must be taken into account to understand its nutritional value and benefits. Similar to other mammals, goat colostrum is the first milk produced after birth. It is rich in immunoglobulins, which provide vital passive immunity to newborns. In addition, goat colostrum contains higher levels of protein, fat, vitamins, and minerals than mature milk, ensuring a strong start to the baby's growth and development. During the early stages of lactation, goat's milk has distinct characteristics. It tends to have higher levels of protein, minerals, and fat than mature milk. The higher fat content provides energy for the growing kid, while the higher protein content contributes to muscle and tissue development. This early lactation milk plays a crucial role in meeting the nutritional needs of the rapidly growing newborn. As lactation progresses, the composition of goat's milk reaches a more stable pattern in mid‐lactation. The levels of protein, fat, lactose, vitamins, and minerals reach a balance suitable for maintaining the growth and nutritional needs of the kid. Mid‐lactation milk is typically characterized by a moderate fat content and a balanced nutrient profile. It serves as a consistent source of nutrition for the growing infant throughout this stage.

Toward the end of lactation, the composition of goat's milk can change further. Late lactation milk may show a gradual decrease in fat content while protein and lactose content remain constant. In addition, variations in mineral content may be observed during this period. Despite these changes, late lactation milk continues to provide essential nutrients for the growing kid. It is important to remember that individual variations in goat milk composition can occur at different stages of lactation due to factors such as breed, diet, and management practices. Understanding these variations is critical to optimizing kid nutrition and ensuring that goat's milk continues to serve as a valuable source of essential nutrients throughout lactation. By recognizing and exploiting the specific composition and properties of goat's milk at each stage, we can ensure the proper growth and development of young kids while benefiting from the nutritional qualities of this unique dairy product (Fekadu et al., 2005).

1. Goat health and welfare: Goat health and welfare can also affect the protein yield and quality of goat's milk. Goats that are stressed or sick may produce milk with a lower protein content. In addition, goats that are not well nourished may also produce milk with a lower protein content. It is therefore important to ensure that goats are healthy and well nourished to ensure a high protein yield and quality of milk. The quality, physicochemical composition, and properties of goat's milk are closely linked to the health status of the lactating animals. Several factors related to goat health can significantly influence milk characteristics. One of the most important factors is the presence of mastitis, an inflammatory condition of the udder. Mastitic milk from goats affected by this condition often shows changes in quality and composition. It may have an increased somatic cell count, reduced milk yield, changes in milk fat and protein content, and elevated levels of inflammatory markers. In addition, mastitic milk may have an undesirable taste, odor, and color, affecting its overall acceptability. Metabolic disorders also play a role in shaping the milk composition and quality of lactating goats. Conditions such as ketosis or acidosis can lead to deviations from the normal milk profile. Goats suffering from these metabolic disorders may exhibit reduced milk fat content, variations in milk pH, and changes in the levels of components such as lactose. These changes in milk composition are the result of disturbed metabolic processes in the goat's body and can affect the taste, nutritional value, and processing properties of the milk. Nutritional imbalances have a direct impact on milk quality in lactating goats. Inadequate or imbalanced nutrition can result in reduced milk yield, altered fat content, and reduced nutritional value. Deficiencies in essential nutrients such as vitamins, minerals, or fatty acids can lead to variations in milk composition. Conversely, excessive intake of certain nutrients, such as excess protein or energy, can also upset the balance of milk constituents and adversely affect milk quality.

Infectious diseases are another major threat to the health of lactating goats and consequently to milk quality. Diseases such as brucellosis or caprine arthritis encephalitis (CAE) can affect the overall health of the animal and affect milk characteristics. Goats suffering from infectious diseases can show changes in milk composition, taste, and safety. In such cases, it is often necessary to discard the milk to prevent the transmission of pathogens through consumption. The stress levels and handling practices of lactating goats can also affect milk quality. High levels of stress caused by factors such as rough handling, environmental disturbance, or social disruption can lead to variations in milk composition and properties. Stress‐related changes in milk composition can include changes in cortisol levels, immunoglobulin content, and fat composition. Minimizing stress and providing a calm and conducive environment for lactating goats can help maintain milk quality. To ensure high‐quality goat milk production, it is vital to prioritize the health and welfare of lactating animals. Regular veterinary care, disease prevention, proper nutrition, and good handling practices are key to maintaining the health of lactating goats. By promoting good health, goat farmers can optimize milk quality, benefiting both goats and consumers of this valuable dairy product (Sevi et al., 2009).

**FIGURE 2:**
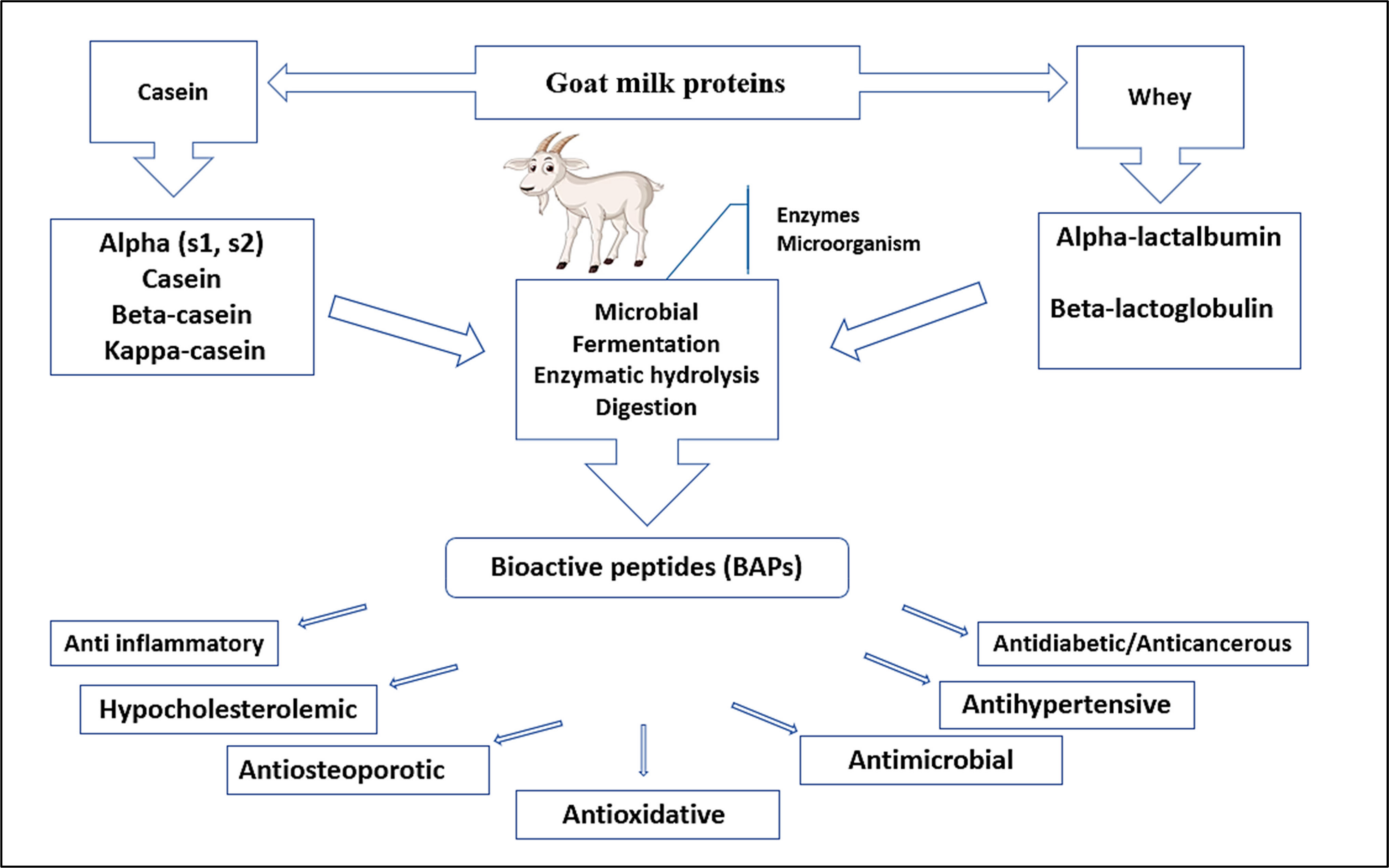
Goat milk proteins and bioactive peptides benefits.

2 Milking and handling practices: Milking and handling practices can also affect the protein yield and quality of goat's milk. Milk that is not collected and handled properly can become contaminated, which can affect the protein content and quality of the milk. It is important to use clean equipment and follow proper milking and handling practices to ensure that the milk is not contaminated (Sandrucci et al., 2019). These factors include goat breed, feeding, stage of lactation, goat health and welfare, and milking and handling practices. By ensuring that these factors are optimal, farmers can produce high‐quality goat's milk with high protein content, which can be used for human consumption or the production of goat milk products. Figure 2 shows the benefits of goat milk proteins and bioactive peptides. Here are some specific examples that illustrate the influence of zootechnical factors on milk quality:

Feed composition: The type and quality of feed given to dairy animals can have a significant impact on milk composition. For example, feeding cows a diet rich in omega‐3 fatty acids, such as linseed, can increase the concentration of omega‐3 fatty acids in their milk. Similarly, providing goats with a diet rich in carotenoids, such as fresh forage, can enhance the yellowish color of their milk due to increased levels of carotenoids.

Grazing systems: The grazing system used for dairy animals can affect milk quality. Dairy cows or goats allowed to graze on diverse pastures with a variety of plant species tend to produce milk with higher levels of beneficial compounds such as polyunsaturated fatty acids and antioxidants. In contrast, animals restricted to limited grazing or fed only stored feed may show differences in milk composition.

Herd health management: The health status of the dairy herd plays a critical role in milk quality. Proper management practices, including vaccination programs, regular veterinary care, and disease prevention measures, can help ensure a healthier herd and reduce the likelihood of milk contamination. For example, maintaining good udder health through proper hygiene practices and mastitis prevention protocols can have a significant impact on milk quality by reducing somatic cell counts and bacterial contamination.

Breed selection: Different dairy breeds have different genetic potentials for milk production and composition. For example, some breeds are known to produce milk with higher fat and protein content than others. Choosing the right breed to meet specific milk quality objectives can have a significant impact on the desired milk characteristics.

Milking techniques: Good milking practices are essential for maintaining milk quality. Factors such as milking hygiene, milking equipment cleanliness, and milking frequency can influence milk composition and minimize bacterial contamination. Adequate training of milkers, regular maintenance of equipment, and adherence to milking protocols are essential to ensure high milk quality.

These examples highlight the influence of zootechnical factors, including feed composition, grazing systems, herd health management, and breed selection and milking techniques, on milk quality. By considering and optimizing these factors, dairy producers can improve the nutritional profile, taste, and safety of milk to meet consumer demands and industry standards.

### 2.2 Coprecipitates of goat milk proteins

Coprecipitation is the simultaneous precipitation of two or more substances from a solution, resulting in the formation of a complex between the precipitants (O'Regan et al., 2009). Coprecipitation is a widely used technique in various industries, including the food, pharmaceutical, and biotechnology industries. In the food industry, coprecipitates of milk proteins are used to enhance the functional properties of milk proteins, making them suitable for a wide range of food applications. In this article, we will discuss goat milk protein coprecipitates, their types, methods of coprecipitation, benefits, and applications.

Coprecipitates of goat milk proteins refer to the complex formed by the simultaneous precipitation of two or more proteins from a goat milk solution. These coprecipitates can contain either casein or whey proteins or both. Casein‐based coprecipitates are formed by mixing casein with another protein such as whey protein, whereas whey protein‐based coprecipitates are formed by mixing whey protein with another protein such as casein. Coprecipitates of goat milk proteins can be divided into two types based on the method of preparation:

1. Chemical coprecipitates: Chemical coprecipitates are formed by adding a chemical reagent to a solution of goat milk proteins. The chemical reagent reacts with the protein molecules resulting in the formation of a complex. Common chemical reagents used for coprecipitation of goat milk proteins include calcium chloride, ammonium sulfate, and sodium chloride. The study characterized the chemical properties of coprecipitates formed by goat milk proteins with iron and calcium. The researchers used various analytical techniques to determine the composition and functional properties of the coprecipitates. The results provided insights into the interactions between goat milk proteins and minerals, which may have implications for the development of functional food products (Deeth & Lewis, 2015).
2. Physical coprecipitates: Physical coprecipitates are formed by the physical mixing of two or more proteins in a solution, followed by precipitation. Physical coprecipitation is often achieved by using a suitable surfactant or by changing the pH of the solution (O'mahony & Fox, 2013).

Coprecipitation of goat milk proteins can be achieved by several methods (AlGhsyar et al., 2018), each of which has its own advantages and disadvantages. Some studies have investigated the formation and characterization of microparticles resulting from the complex coacervation of goat milk proteins and gum arabic. Researchers investigated the physicochemical properties, particle size distribution, and morphology of the coprecipitates. They also evaluated the effect of pH, protein/gum arabic ratio, and other factors on the formation and stability of the microparticles. The results shed light on the potential applications of these coprecipitates in the food and pharmaceutical industries. Some of the commonly used methods are as follows:

1. Salt coprecipitation: Salt coprecipitation involves the addition of a salt to a solution of goat milk proteins to induce precipitation. The salt reduces the solubility of the proteins leading to the formation of a complex. The advantages of salt coprecipitation are that it is a simple and inexpensive method. The study investigated salt‐induced coprecipitation of goat milk proteins with calcium phosphate. The researchers studied the effects of different salts, including sodium chloride and calcium chloride, on the coprecipitation process and the resulting protein structures. They used various techniques to analyze the coprecipitates, including electrophoresis, turbidity measurements, and scanning electron microscopy. The results provided insights into the interactions between goat milk proteins and salts, which are important for understanding the functionality of these coprecipitates in food and dairy applications (Yen et al., 2015).
2. Acid coprecipitation: In acid coprecipitation, an acid is added to a solution of goat milk proteins to induce precipitation. The acid causes the proteins to denature, resulting in the formation of a complex. The advantages of acid coprecipitation are that it is a simple and rapid method. The study investigated acid coprecipitation of goat milk proteins with calcium phosphate and the influence of pH and calcium concentration on the coprecipitation process. The researchers studied the changes in protein structure, solubility, and composition during coprecipitation using various analytical techniques. The results showed the influence of pH and calcium concentration on the coprecipitation efficiency and the resulting properties of the coprecipitates. These findings may contribute to the development of novel dairy‐based products and formulations (Li et al., 2020).
3. Enzyme coprecipitation: Enzyme coprecipitation is a technique that uses an enzyme to cause precipitation in a solution of goat milk proteins. By breaking down the protein molecules, the enzyme forms a complex. This method has the advantage of being gentle and selective. The study investigated enzyme‐mediated coprecipitation of goat milk proteins using transglutaminase. The researchers studied the effect of different reaction conditions, such as enzyme concentration, pH, and incubation time, on the coprecipitation process. They evaluated the changes in protein properties such as solubility, particle size, and rheological behavior after coprecipitation. The results demonstrated the potential of transglutaminase as an enzyme for modifying the functional properties of goat milk proteins by coprecipitation.
4. Thermal coprecipitation: Thermal coprecipitation is a technique in which a solution containing goat milk proteins is heated to induce precipitation. This method is advantageous because it is both economical and uncomplicated. The study investigated thermal coprecipitation of goat milk proteins and starch and evaluated its effect on particle characteristics and rheological properties. The researchers investigated the influence of various parameters such as protein/starch ratio, heating temperature, and heating time on the coprecipitation process. They analyzed the morphology, size distribution, and rheological behavior of the resulting coprecipitates. The results provided insights into the thermal coprecipitation process and its potential for modifying the functional properties of goat milk proteins in food systems (Gawande et al., 2022).

### 2.3 Nutritional properties of goat milk proteins

According to Park (2010), although goats provide a small proportion of the world's milk supply, their impact on human welfare is significant. Goat's milk has long been used for human consumption, as noted by Arrichiello et al. (2022). Prosser (2021) points out that several research studies have been conducted to explore the nutritional value and technological properties of goat's milk for the production of consumer products such as cheese, yogurts, and UHT milk.

**FIGURE 3:**
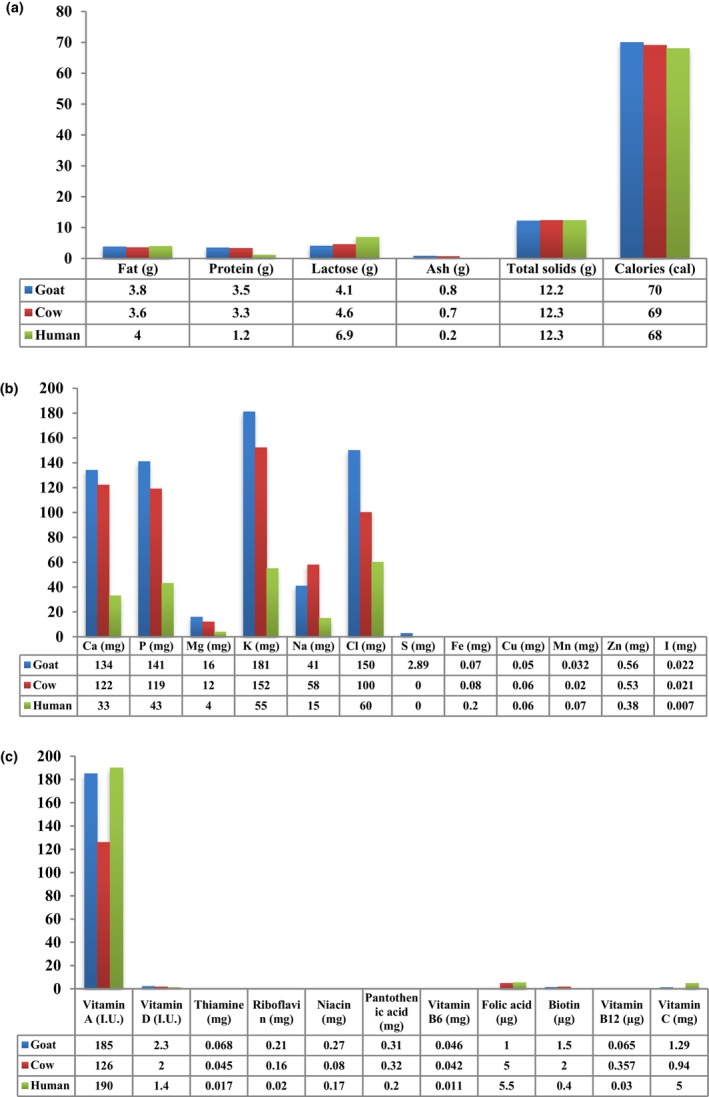
Average concentrations (per 100 g) of (a) Basic nutrients, (b) Minerals, and (c) Vitamins in goat's milk compared with those in cow's and human Milk (Park, 2010).

According to Rai et al. (2022), goat's and cow's milk have a similar basic composition. However, Park (2010) reports that goat's milk contains an average of 12.2% total solids, consisting of 3.8% fat, 3.5% protein, 4.1% lactose, and 0.8% ash. Compared to cow's milk, goat's milk is higher in fat, protein, and ash and lower in lactose. Compared to cow's milk, goat's milk has higher levels of medium‐chain fatty acids such as caprylic acid (C8) and capric acid (C10). On the other hand, cow's milk contains higher levels of butyric acid (C4) and occasionally palmitic acid (C16:0). As a result, the regulation of mammary cells differs between goats and cows, particularly in the elongation process of fatty acids newly synthesized by the “fatty acid synthase” complex. Studying and comparing the mechanisms in these two species would improve our understanding of the regulation of milk fat synthesis in ruminants. This area of knowledge is less explored in ruminants than in rodents (Chilliard et al., 2003). In addition, Delger (2021) points out that goat's milk has slightly lower total casein content but higher nonprotein nitrogen content than cow's milk. Saikia et al. (2022) state that the protein and ash content of goat's and cow's milk is three to four times higher than that of human milk. However, the total solids and calorie contents of goat's milk, cow's milk, and human milk are comparable. Figure 3 illustrates the typical concentrations (per 100 g) of essential nutrients, minerals, and vitamins found in goat's milk, cow's milk, and human milk, allowing a comparison between the three.

Getaneh et al. (2016) highlighted that goat's milk has special characteristics that distinguish it from cow's milk, such as better digestibility, higher alkalinity, and stronger buffering capacity. Furthermore, according to Singh et al. (2021), goat's milk is a preferred choice for individuals with cow's milk allergy and lactose intolerance. Meanwhile, Sumarmono (2022) highlights that genetic variations result in differences in the proportions of the four essential caseins (αs‐1, αs‐2, β, and κ) and the size of micelles in goat's milk. Grappin and Jeunet (1979) found that goat's milk has a higher dye‐binding capacity per unit protein (1% higher than cow's milk) and a lower infrared absorption (4% lower than cow's milk). As a result, different calibration curves are required for each species to assess milk protein content. Kumar et al. (2012) verified these results, demonstrating that the use of bovine milk standards to test goat's milk resulted in a reduction of 0.04% in fat and 0.27% in protein. Although the primary classifications of α‐, β‐, κ‐caseins, β‐lactoglobulin, and α‐lactalbumin are similar in goat's milk and the major cow milk proteins, there are significant differences in genetic variations and their frequencies in goat populations (Kumar et al., 2012). Recent studies have focused on αs1‐casein, which has six distinct variants (A, B, C, E, F, and “null”) in goat's milk. Unlike cow's milk, where αs1‐casein is the main αs‐casein, some goat's milk lacks the protein responsible for certain types of coagulation due to the absence or “null” type of αs1‐casein. This can affect the cheese‐making process. The weaker gel consistency of goat's milk yogurt compared to cow's milk yogurt can be attributed to its low casein content, α‐casein ratio, and micellar size (Sumarmono, 2022). According to Fan et al. (2021), goat's milk has lower thermal stability compared to cow's milk due to its high ionic calcium content and low micellar solvation, resulting in thermal instability. Genetic variations in goat milk proteins result in differences in the proportions of α‐s1‐ and α‐s2‐caseins between goat individuals and breeds, as highlighted by Sumarmono (2022). Furthermore, Da Silva and da Costa (2019) report that goat milk products have different textural properties, such as lower hardness, consistency, adhesiveness, extrusion force, stability, and higher syneresis than cow milk products. Domagała (2009) also mentions that goat milk products have inferior textural properties compared to cow milk products. Goat's milk typically contains oligosaccharides in the range of 250 to 300 mg/L, which is about 4 to 5 times higher than cow's milk and 10 times higher than sheep's milk. However, it is still considerably lower than the oligosaccharide content of human milk, which ranges from 5 to 8 g/L. The oligosaccharides present in goat's milk are structurally complex and have a profile more similar to human milk than cow's or sheep's milk. High yields of these oligosaccharides have been successfully isolated from goat's milk (Martinez‐Ferez et al., 2006) and research has demonstrated their anti‐inflammatory properties in rat models of colitis induced by hapten or dextran sodium sulfate (Daddaoua et al., 2006). As a result, goat's milk is considered a promising natural source of oligosaccharides similar to those found in human milk, making it an attractive option for the development of infant formulae, follow‐on formulae, and health‐promoting products due to its composition and content.

### 2.4 Functional properties of goat milk proteins

Goat milk proteins (GMPCs) have been found to have various applications in the food industry due to their unique properties, including emulsification, gelling, and water binding. In this article, we will discuss some of the applications of GMPCs in the food industry (Alu'datt et al., 2013).

#### 2.4.1 Emulsification

One of the key properties of GMPCs is their ability to form stable emulsions. GMPCs can be used as emulsifiers to stabilize oil‐in‐water emulsions in various food products such as salad dressings, mayonnaise, and cream. The emulsifying properties of GMPCs are attributed to the presence of whey proteins, which have a high surface activity and can adsorb at the oil–water interface. In addition, casein proteins in GMPCs can form a network structure around the dispersed oil droplets, further stabilizing the emulsion (Li et al., 2020). Ni and Raikos (2019) investigated the effect of lactic acid bacterial fermentation on an oil‐in‐water emulsion stabilized with goat milk proteins. The researchers used optical microscopy and Turbiscan analysis to monitor changes in the microstructure and droplet size of the emulsion during fermentation. The results showed that pH‐driven effects caused conformational changes in the milk proteins, as indicated by the determination of total thiol groups. These changes also affected the composition of the protein interface. At pH levels below 5.5 and temperatures above 37.9°C, there was an increase in the average droplet size, which was attributed to flocculation phenomena resulting from reduced electrostatic repulsion. During the early stages of fermentation (1–3 h), casein solubility increased but precipitated at pH values below 4.8. LC–MS/MS analysis confirmed that caseins (αs1‐, αs2‐, β‐, and κ‐casein) were the predominant protein species present at the oil–water interface at the end of the fermentation process. These caseins contributed to the stability of the emulsion by reducing the droplet diameter.

#### 2.4.2 Gelation

GMPCs can also form gels when heated in the presence of calcium ions. The gelling properties of GMPCs are attributed to the presence of casein proteins, which can form a network structure when heated in the presence of calcium ions (Deeth & Lewis, 2015). This network structure can trap water and other components, resulting in a gel‐like texture. GMPCs can be used as gelling agents in a variety of foods such as cheese, yogurt, and puddings.

#### 2.4.3 Water binding

GMPCs are also known for their ability to bind water (Fox et al., 2015). This property makes them useful as a functional ingredient in meat products, where they can improve water retention and increase yield. GMPCs can also be used as binders in vegetarian and vegan products to improve texture and binding properties.

#### 2.4.4 Nutritional Supplements

GMPCs are also used as a source of high‐quality protein in dietary supplements. GMPCs are rich in essential amino acids (Du et al., 2022), making them valuable ingredient for individuals with high protein requirements, such as athletes and bodybuilders. In addition, GMPCs are low in lactose, making them suitable for individuals with lactose intolerance (Qin et al., 2021).

#### 2.4.5 Infant Formula

GMPCs can also be used as a source of protein in infant formulae. Goat's milk is known for its high nutritional value (Nayik et al., 2022) and GMPCs can provide a good balance of casein and whey proteins for infant formula. In addition, GMPCs are low in allergenic proteins, making them a suitable alternative for infants with cow milk protein allergy. Zhou et al. (2014) found that the developmental and blood indicators of nutritional status of infants fed a whole goat's milk‐based infant formula were similar to those of infants fed a regular cow's milk formula supplemented with whey. The fact that there were no notable differences between the groups of infants fed different formulae in terms of various health‐related outcomes and the occurrence of serious adverse events suggests that the use of goat's milk in infant formula is safe (Al‐Saadi et al., 2014).

#### 2.4.6 Dairy Alternatives

GMPCs can also be used as a functional ingredient in dairy alternatives such as plant‐based milks and cheeses. GMPCs can improve the texture and mouthfeel of these products, making them more similar to their dairy counterparts. Gursel et al. (2016) showed that a variety of milk protein‐based products can be used to produce goat milk yoghurt with enhanced physical and sensory properties. Yogurts fortified with either NaCn or yogurt texture improver (YTI) exhibited a denser structure and reduced whey separation compared to yogurt fortified with whey protein concentrates (WPC). The addition of WPC or WPI resulted in higher acetaldehyde levels in goat's milk yogurt. However, the use of WPI did not improve the texture of the yogurt. Further research is needed to determine the ideal CN:whey protein ratio in goat's milk yogurt, especially when fortified with whey protein‐based ingredients.

#### 2.4.7 Other Applications

GMPCs have also been used in other applications, such as the production of biodegradable films and coatings for food packaging (Al‐Saadi et al., 2014). GMPCs can form a film around food products, providing a barrier to oxygen and moisture, which can improve shelf life.

### 2.5 Health benefits of goat milk proteins

Goat's milk is a nutritious food, rich in proteins, vitamins, minerals, and other essential nutrients (Yadav et al., 2016). In particular, the proteins found in goat's milk have been shown to have numerous health benefits. Goat's milk is a nutritious food, rich in proteins, vitamins, minerals, and other essential nutrients (Yadav et al., 2016). In particular, the proteins found in goat's milk have been shown to provide numerous health benefits. In this response, we will explore the various health benefits of goat milk proteins, including their immunomodulatory effects, allergy management properties, anti‐inflammatory and antioxidant effects, antimicrobial and anticancer properties, and potential therapeutic applications.

## 3 IMMUNOMODULATORY EFFECTS AND ALLERGY MANAGEMENT

Goat milk proteins have been shown to have immunomodulatory properties that can help improve the function of the immune system. Research has shown that consumption of goat's milk can help regulate the immune response and improve the body's ability to fight infection and disease. Goat's milk is an important component in many biological reactions and can have anti‐inflammatory and antioxidant effects on the body (O'Shea et al., 2004). Inflammation is the body's primary response to infection and oxidation has been implicated in the development of several diseases, including cancer. Goat's milk also contains probiotics and prebiotics, which are necessary for maintaining a healthy gut microflora and protecting against the negative effects of pathogenic infections and allergies.

While goat's milk may not be an ideal substitute for people allergic to cow's milk, recent studies suggest that it has immunomodulatory properties in both in vitro and human studies. Current research has investigated how goat's milk affects human blood cells and found that it can stimulate the release of NO and induce the production of cytokines (such as IL‐10, TNF, and IL‐6). The release of NO may be beneficial for cardiovascular health in those who consume goat's milk and may also have antibacterial properties which may help to prevent infection (O'Shea et al., 2004).

In addition, goat milk proteins may help to manage allergies such as cow's milk allergy due to their unique protein composition. Goat milk proteins contain lower levels of α‐s1‐casein, the protein responsible for most allergic reactions to cow's milk. Studies have shown that goat's milk is superior to cow's milk in relieving symptoms of colic, minor digestive problems, asthma, and eczema. For infants and children who are sensitive to cow's milk, the administration of goat's milk has been shown to be effective in 30%–40% of cases, and in some cases, even higher (e.g., in one particular study, 49 of 55 children treated showed improvement) (Haenlein, 2004; McCullough, 2003). Lactose intolerance occurs when the body lacks the enzyme lactase, which is needed to break down the lactose in milk. As a result, undigested lactose passes into the large intestine where it is fermented by microbes, resulting in the production of gases such as methane, hydrogen, carbon dioxide, and short‐chain fatty acids, which cause gastrointestinal problems such as diarrhea, abdominal pain, and bloating (Russell et al., 2011). It is important to distinguish lactose intolerance from cow's milk allergy, which is an immune reaction to the proteins in cow's milk.

## 4 ANTI‐INFLAMMATORY AND ANTIOXIDANT EFFECTS

Goat milk proteins also have anti‐inflammatory and antioxidant properties (Ahmed et al., 2015; Jirillo & Magrone, 2014). Several studies have shown that goat milk proteins have anti‐inflammatory effects, which may benefit people suffering from chronic inflammatory conditions such as arthritis, asthma, and allergies. Goat's milk contains several types of protein, including caseins, whey proteins, and immunoglobulins. Of these, the most abundant protein in goat's milk is casein, which makes up around 80% of the total protein content. Caseins are a group of proteins that are highly resistant to digestion, making them a slow‐release source of protein that can provide sustained energy and satiety.

Research has shown that certain types of casein, such as α‐casein, have anti‐inflammatory properties. Alpha‐casein is a potent inhibitor of angiotensin‐converting enzyme (ACE), which plays a role in regulating blood pressure and inflammation. By inhibiting ACE, α‐casein may reduce inflammation and potentially lower the risk of cardiovascular disease. Another protein in goat's milk with anti‐inflammatory properties is lactoferrin (Tillib et al., 2014). Lactoferrin is an iron‐binding protein found in milk, saliva, tears, and other body fluids. Studies have shown that lactoferrin can modulate the immune response, inhibit the growth of bacteria and viruses, and reduce inflammation. Lactoferrin has been shown to be effective in reducing inflammation in animal models of arthritis, colitis, and other inflammatory conditions (Tillib et al., 2014). Goat's milk also contains whey proteins, which have been shown to have anti‐inflammatory effects (Zhao et al., 2023). Whey proteins contain several bioactive peptides (Ahmed et al., 2015), which have been shown to modulate the immune system, reduce inflammation, and improve gut health. Ahmed et al. (2015) confirmed that goat's milk caseins and whey proteins contain peptide fractions and crude pepsin hydrolyzates that exhibit potent scavenging activities against superoxide and DPPH radicals. The researchers identified the specific amino acid residues and motifs, particularly those with Leu and Ser–Leu/Thr–Leu/Pro–Leu sequences at the ends of the peptides that contribute to their superior antioxidant properties. This study is the first to demonstrate the production of antioxidant peptides from separated whey and casein fractions of goat's milk using pepsin hydrolysis, which offers a viable alternative to waste utilization in the cheese industry. These goat protein fractions have potential applications in the nutraceutical industry as bioactive peptides and could be promising candidates for therapeutic use in the pharmaceutical industry for the treatment or prevention of oxidative stress‐related diseases (Ahmed et al., 2015).

## 5 ANTIMICROBIAL AND ANTICANCER PROPERTIES

Goat milk proteins also exhibit antimicrobial and anticancer properties (Atanasova & Ivanova, 2010; Dhasmana et al., 2022), and fresh goat's milk and fermented goat's milk offer potential health benefits due to the presence of bioactive compounds in these products (Hammam et al., 2021). Milk proteins have been shown to be capable of producing antimicrobial peptides, of which lactoferrin‐derived peptides are the best known. Lactoferrin not only is primarily responsible for iron transport but also plays a role in several physiological functions, such as regulating iron absorption and immune responses. It has antioxidant, anti‐inflammatory, and anti‐carcinogenic properties, but its antimicrobial capabilities have been extensively studied (García‐Montoya et al., 2012). In addition, αs2‐casein degraded by pepsin, a gastrointestinal enzyme, has also been found to have antimicrobial activity (Park, 2009a, 2009b). Based on the results of this study, it appears that *Bifidobacterium longum* Bb‐46 grows more effectively in goat's milk than in cow's milk (Horáčková et al., 2015; Slačanac et al., 2010).

Some studies have suggested that goat milk proteins may have anticancer properties and may be effective in preventing the growth and spread of cancer cells, with applications in conditions such as asthma, osteoporosis, and cardiovascular disease. The protein profile of goat whey was investigated by Medeiros et al. (2018) to assess its potential effects on oxidative stress, bacteria, tumors, and human red blood cells in vitro. The method involved skimming goat whey to obtain a crude protein extract (CPE), which was then subjected to ammonium sulfate precipitation to obtain protein fractions (F). The CPE showed antioxidant properties while the protein fractions did not. In cytotoxicity tests performed on rat C6 glioma cells, the CPE caused a significant tumor cell death rate of over 70% when used at concentrations of 0.05 and 0.1 μg/mL. The results of this study suggest that CPE has the potential to be an effective compound with antioxidant, bacteriostatic, and cytotoxic properties against tumor cells, which was demonstrated for the first time in this study (Medeiros et al., 2018). Finally, the following Table 1 shows the health benefits of goat's milk.

**TABLE 1:** Goat's milk health effects.

Health effect	Study results	References
Cytomodulatory effect	Evidence suggests that caseinophosphopeptides (CPPs) have cytomodulatory characteristics, either by inhibiting the growth of cancer cells or by enhancing the activity of immune‐competent cells.	Meisel and FitzGerald (2003)
Increase mineral bioavailability	Minerals: Bioavailability of selenium, zinc, and copper in goat‐milk‐fed rodents was higher than in cow‐milk‐fed rats.	Alférez et al. (2003), Barrionuevo et al. (2003)
Anti‐inflammation	Oligosaccharide: Research in rats shows that they help reduce gut inflammation and assist in the healing of injured digestive epithelium.	Lara‐Villoslada et al. (2006)
Prebiotic antipathogenic effect	Oligosaccharide: Goat's milk, thanks to the oligosaccharides it contains, can be used as an addition in milk composition and has been linked to antipathogenic and probiotic benefits as well as improvement of the central nervous system.	Meyrand et al. (2013), Kunz and Rudloff (2006)
Enhanced mineral uptake	Minerals: A meal rich in goat's milk reduced anemia and increased iron storage in key tissues in rodents.	Alférez et al. (2006)
Energy providing effect	Goat's milk has a significant impact on providing energy, especially to growing children, due to the medium‐chain triglyceride content (MCT) it contains.	Park and Haenlein (2008)
Essential for metabolic processes	Cholesterol: Both bile acid and vitamin D use it as a biochemical precursor. DNA production, lipid transfer, and cell division all rely on it for their respective biochemical functions.	Park (2009a, 2009b)
Cell protection activity	Oligosaccharide: They promote the growth of *Lactobacillus bifidus* in the intestine, which aids in the defense of gut mucous cells against diseases, especially in newborns.	Raynal‐Ljutovac et al. (2008)
Antagonistic or agonistic activity	Opioid peptides, like those found in β‐ and α‐casein, are recognized for their antagonism or agonistic actions.	Park (2009a, 2009b)
Hypocholesterolemia	By preventing cholesterol from being dissolved and deposited in gallstones, the fatty acids present are known to have a hypocholesterolemic impact on the blood and tissue.	Park (2017)
Immunomodulatory effect	Lymphocyte growth, antibody formation, and cytokine regulation are just some of the immunomodulatory impacts of peptides and protein hydrolyzates isolated from main whey proteins and milk caseins.	Turkmen (2017)
Antiappetizing effect	Cholesterol and cholecystokinin synthesis are both influenced by the overall amount of whey protein in the food. (Appetite‐suppressing hormone.)	Turkmen (2017)
ACE inhibitors	Four new ACE inhibitory peptides were discovered in the termolisin‐generated caprine (β‐Lg) hydrolyzate.	Park (2017)
Antihypertensive and immunostimulating	Peptides with immunostimulatory and antihypertensive properties can be synthesized using ‐casein from caprine milk.	Ibrahim et al. (2017)
Angiotensin‐converting enzyme inhibitors (ACE inhibitors)	Caprine milk contains β‐casein f58–65 and αs2‐casein f182–187, both of which are candidates for the production of ACE inhibitory peptides.	Park (2017)
Anticariogenic effects	Calcium‐binding casein phosphopeptides (CPP) in the mouth have anticariogenic qualities, helping to stop caries ulcers from developing by recalcifying the tooth enamel and blocking calcium absorption by bacteria that cause oral plaque.	Park (2017)
Antioxidant property	Peptides synthesized from αs‐casein have been shown to scavenge free radicals and protect against lipid degradation caused by enzymatic and nonenzymic alike.	Park (2017)
Antioxidant property	The antioxidant glutathione is crucial for cellular defense and healing, and it is present in higher concentrations of whey protein that has been treated at moderate temperatures.	Park and Haenlein (2017)
Increase in overall nutrition	Minerals: The health and well‐being of rats given GM was significantly improved, with increased liver weights, increased hemoglobin iron concentration, and improved iron intake.	Park (2017)
Allergy	Minerals: Some babies with gut sensitivities improved after receiving goat's milk during birth and nursing.	Park and Haenlein (2017)
Promotion of growth factors	Minerals: Caprine milk was tested for the presence of epidermal growth factor (EGF) using a human epidermal growth factor (hEGF) monoclonal antibody, but the quantity of growth factors was considerably lower than in GM.	Clark and Mora García (2017)
Fat absorption	Phospholipids help with fat intake because they create a protective layer around fat globules, allowing for more even distribution of the fat. Phospholipids facilitate fat export from the liver via a process known as lipotropic action.	Park (2017)
Antimicrobial activity	Antimicrobial activity of a lactoferrin‐derived peptide (Lactoferricins) has been demonstrated against a wide range of Gram‐negative and ‐positive bacteria, fungus, and yeast.	Giraldo et al., 2017, Niaz et al. (2019)
Antioxidant property	The antioxidant properties of cultured GM with *Lactobacillus fermentum* (M4) were particularly impressive.	Panchal et al. (2020)
Antidiabetic	Goat's milk contains casein hydrolyzates, which may help reduce insulin intolerance and make current treatments for type 2 diabetes more effective.	Gong et al. (2020)
Antithrombotic activity	Human platelet clumping was reduced when treated with caprine κ‐CMP or its protease hydrolyzates.	Cakir and Tunali‐Akbay (2021)
Antiviral property	Preliminary data suggest a casein portion component mediates the nonspecific process. This portion may contain one or more components that engage with viral capsid receptors or membranes to inhibit cell entry and propagation of pseudo‐virus SARS‐CoV‐2, Coxsackievirus A9, and HSV‐1.	Rubin et al. (2021)
Antiviral property	The advantages of goat milk peptides as IL‐6 antagonists have been investigated in this research, and the results suggest that this approach may be useful for preventing the excess of IL‐6 and managing COVID‐19.	Bhavaniramya et al. (2022)
Gut inflammation reduction	This study is the first to test goat mEVs on an in vitro GI inflammatory model. Goat mEVs may regulate inflammation and benefit the gut lining.	Mecocci et al. (2022)
Immunomodulatory effect	Human milk contains glycosphingolipids (GSLs), which aid in immunological regulation, promote digestive development, and protect against gastrointestinal infections. Goat's milk contained a total of 33 gangliosides, including 4 GB and 23 previously unreported gangliosides. Human milk predominantly contained GM1, while cattle and goat's milk primarily contained GD3 and GM3, respectively; N‐acetylneuraminic acid (Neu5Ac) was identified in >88% of GSLs in cow's and goat's milk. These findings will aid in the creation of specialized baby formulae based on human milk, which is important because various GSLs have varying health advantages.	Li et al. (2023)
Improve digestion	Baby formula made from goat's milk had a higher relative prevalence of the intestinal microbes Blautia, Roseburia, Alistites, and Muribaculum, according to 16S rRNA analysis. This research adds to our understanding of the gut microecology effects of goat's milk‐based baby feeds, and may lead to their wider use.	Chen et al. (2023)
Antioxidant property	The antioxidant capacity and approximated in vitro digestibility of ingested goat milk products may be improved by repetitive freeze–thaw processes.	Ma et al. (2023)

Unlike cows and ewes, goat's milk presents a challenge when it comes to interpreting somatic cell counts in relation to potential infections. To gain a better understanding of this relationship, Sánchez‐Macías et al. (2013) conducted a study in which they produced goat milk cheeses with different somatic cell counts. Their results showed that the quality of the cheeses depended on the amount of these compounds. Similarly, Raynal‐Ljutovac et al. (2007) investigated the effect of somatic cell count on milk composition, suitability for cheese production, and overall quality of dairy products made from goat's and sheep's milk. They concluded that stricter control of somatic cell counts in milk from small ruminants is necessary to ensure the safety of dairy products derived from this type of milk.

## 6 CONCLUSION, FUTURE RESEARCH DIRECTIONS AND IMPLICATIONS

### 6.1 Conclusion

Goat milk proteins have been shown to have coprecipitates, nutritional and functional properties, and health benefits. The coprecipitates of goat milk proteins have been shown to have enhanced solubility and emulsifying properties compared to single protein isolates. Goat milk proteins are highly nutritious, containing all the essential amino acids required by the human body, as well as a high concentration of minerals and vitamins. The functional properties of goat milk proteins make them useful in the food industry for various applications, including improving the texture, emulsification, and foaming properties of food products. In addition, studies have shown that the proteins found in goat's milk have health benefits, such as anti‐inflammatory and immunomodulatory effects. As a result, these proteins are considered a potential therapeutic option for a variety of diseases.

### 6.2 Future research

Despite the growing interest in goat milk proteins, more research is needed to fully understand their properties and potential applications. Further research in this area could focus on assessing the impact of processing techniques on the functional and nutritional properties of goat milk proteins. In addition, the potential use of goat milk proteins in the development of functional foods, nutraceuticals, and pharmaceuticals could be investigated. Further research could also investigate the potential use of goat milk proteins in the treatment of inflammatory and immune‐mediated diseases and as a source of bioactive peptides with potential health benefits.

The results of this review have several implications for the food and health industries. Goat milk proteins have potential applications in the food industry as an alternative to cow milk proteins due to their functional and nutritional properties. The high solubility and emulsifying properties of goat milk protein coprecipitates make them attractive for use in a wide range of food products. In addition, the health benefits of goat milk proteins, such as anti‐inflammatory and immunomodulatory effects, make them a promising therapeutic option for various diseases. The development of functional foods and nutraceuticals based on goat milk proteins could have a significant impact on the prevention and treatment of various diseases. Overall, the results of this review suggest that goat milk proteins have significant potential for use in the food and health industries and warrant further investigation.

## AUTHOR CONTRIBUTIONS

**Qausar Hamed ALKaisy:** Conceptualization (equal); investigation (equal); resources (equal); writing – original draft (equal). **Jasim S. Al‐Saadi:** Conceptualization (equal); investigation (equal); writing – original draft (equal). **Ali khudhair Jaber AL‐Rikabi:** Conceptualization (equal); investigation (equal); writing – original draft (equal). **Ammar B. Altemimi:** Conceptualization (equal); writing – review and editing (equal). **Mohammad Ali Hesarinejad:** Conceptualization (equal); writing – review and editing (equal). **Tarek Gamal Abedelmaksoud:** Conceptualization (equal); writing – review and editing (equal).

## FUNDING INFORMATION

This research did not receive any specific grant from funding agencies in the public, commercial, or not‐for‐profit sectors.

## CONFLICT OF INTEREST STATEMENT

The authors declare that they have no known competing financial interests or personal relationships that could have appeared to influence the work reported in this paper.

## ETHICS APPROVAL AND CONSENT TO PARTICIPATE

This article does not contain any studies with human or animal subjects.

## CONSENT FOR PUBLICATION

All authors have read and agreed to the published version of the manuscript. All authors read and approved the final manuscript.

## Data Availability

All data generated or analyzed during this study are included in this published article.
